# Prognostic impact of early adjunctive corticosteroid therapy in non-HIV oncology or haematology patients with Pneumocystis jirovecii pneumonia: A propensity score analysis

**DOI:** 10.1371/journal.pone.0250611

**Published:** 2021-04-22

**Authors:** Mehdi Assal, Jérôme Lambert, Laurent Chow-Chine, Magali Bisbal, Luca Servan, Frederic Gonzalez, Jean Manuel de Guibert, Marion Faucher, Norbert Vey, Antoine Sannini, Djamel Mokart

**Affiliations:** 1 Intensive Care Unit, Hôpital La Timone, Marseille, France; 2 Biostatistics Department, Saint Louis Teaching Hospital, Paris, France; 3 Intensive Care Unit, Institut Paoli-Calmettes, Marseille, France; 4 Department of Haematology, Institut Paoli-Calmettes, Marseille, France; Ohio State University Wexner Medical Center Department of Surgery, UNITED STATES

## Abstract

**Purpose:**

While early adjunctive corticosteroid therapy (EACST) has been proven effective in HIV patients with Pneumocystis Jirovecii Pneumonia (PJP), data remains controversial concerning non-HIV oncology or haematology patients.

**Methods:**

This retrospective study included cancer patients without HIV and with diagnosis of PJP admitted in a cancer referral centre, from January-1-2010 to March-31-2017. We compared 30-day and 1-year mortality rate, change in the respiratory item of the Sequential Organ Failure Assessment score(SOFA-_resp_ worsening), use of tracheal intubation between day-1 and day-5 of anti-pneumocystis therapy and occurrence of coinfections between patients with EACST and those with no or late corticosteroid therapy, using an inverse probability weighting propensity score-based (IPW) analysis.

**Results:**

133 non-HIV oncology or haematology PJP patients were included (EACST n = 58, others n = 75). The main underlying conditions were haematological malignancies (n = 107, 80,5%), solid tumour (n = 27, 20,3%) and allogeneic stem cell transplantation (n = 17, 12,8%). Overall 30-day and 1-year mortality rate was 24,1% and 56,4%, respectively. IPW analysis found no difference on 30-day (HR = 1.45, 95% CI [0.7–3.04], p = 0.321) and 1-year (HR = 1.25, CI 95% [0.75–2.09], p = 0.39) mortality rate between groups.

**Conclusion:**

No difference in SOFA-_resp_ worsening, tracheal intubation and coinfections was found between groups. Combination of EACST with anti-pneumocystis therapy in non-HIV onco-haematology PJP-patients was not associated with clinical improvement.

## Introduction

Pneumocystis Jirovecii pneumonia (PJP) is a life-threatening opportunistic infection in immunocompromised patients, caused by the fungus Pneumocystis Jirovecii. Historically, PJP affects mostly patients with human immunodeficiency virus (HIV). However, prevalence of patients with HIV-related PJP has decreased with the use of highly active antiretroviral therapy and routine PJP prophylaxis [1,2]. In contrast, over the last years, advances in cancer treatments (new drugs, intensive chemotherapy, allogeneic stem cell transplantation) resulted in an increased incidence and mortality of PJP among immunocompromised patients without HIV [3–9]. Prior studies showed that mortality rate, due to PJP, is higher in the non-HIV patients than in the HIV-patients [3,6]. In HIV-positive patients with moderate to severe PJP, evidence derived from a meta-analysis on six randomized controlled trials suggests a survival benefit of adjunctive corticosteroids therapy [10,11]. Thereby, adjunctive corticosteroids are strongly recommended by current guidelines in PJP-related patients with HIV [12]. Nevertheless, the clinical benefit of early adjunctive corticosteroid therapy (EACST) in non-HIV PJP-patients remains unclear. Although several observational studies have been published, the clinical improvement of PJP by EACST is still controversial. No randomised controlled trial has yet been conducted to address this issue. Considering the paucity of data on this topic, the primary objective of our study was to assess the efficacity of EACST in addition to anti pneumocystis therapy, on 30-days and 1-year mortality in non-HIV onco-haematology PJP-patients. Secondary objectives were respiratory outcome between day 1 and day 5, need of tracheal intubation at day-5 and occurrence of bacterial, viral and fungal coinfections.

## Methods

### Study population

We conducted a retrospective observational single-center study in our institution, Institut Paoli Calmette, a cancer referral center. The recruitment period was from January-1-2010 to March-31-2017, the follow-up period stood until March-31-2018 and the data collection period was from January-5-2019 to May-31-2019. Inclusion criteria were: adult patients with PJP diagnosis, treated with anti-pneumocystis therapy and admitted in our institution (ICU and/or ward). We excluded from the study patients with documented HIV infection and those who did not receive anti-Pneumocystis therapy. All data were extracted from ICU and hospital information systems. The study was approved by the Paoli-Calmettes Institute Institutional Review Board (Jirove-CORT-IPC 2019–003) which waived the need for informed consent. The methodology adheres to the STROBE statement [13].

### PJP diagnosis

The diagnosis confirmation of PJP was based on the polymerase chain reaction (PCR) positivity from a respiratory specimen, associated with typical respiratory symptoms as dyspnea, cough and fever, and typical findings on Computed Tomography (CT) [14]. In addition, we retrospectively used the Pneumocystis Jivorecii Pneumonia score (PJP-score) in order to eliminate or discuss contentious cases specifically for patients with haematological malignancies. Indeed, this diagnostic score established by Azoulay et al is a multivariable prediction model for PJP diagnosis in haematology patients. This score > 3 had 86,7% sensitivity and 67,7% specificity for PJP and a negative predictive value of 98% [15].

### Definitions and data collections

For all patients the anti-pneumocystis treatment could be started as soon as PJP was suspected (from the day of respiratory sampling to the first day of diagnosis) but initiation of this treatment was left at the discretion of attendant physician. In our institution, sulfamethoxazole/trimethoprim (SMT) is the first line anti-pneumocystis therapy. However, in case of treatment failure or allergic reaction, SMT could be replaced by atovaquone or pentamidine. We defined as “day 1” the first day of PJP diagnosis. At our institution (ward and/or ICU), adjunctive corticosteroid treatment was let at the discretion of the attendant physician. EACST was defined as corticosteroids administration within 48 hours of anti-pneumocystis therapy initiation. We recorded all different types of steroid treatments, daily doses and duration of therapy during hospital stay. Corticosteroid doses were expressed in daily prednisone equivalents [16]. Patients clinical and demographic characteristics are summarized in **Table 1.** Criteria for acute respiratory failure were defined as arterial oxygen saturation lower than 90% or an arterial oxygen pressure (P_a_O_2_) of less than 60 mmHg breathing room air, combined with severe dyspnea at rest. We daily used respiratory item of the Sequential Organ Failure Assessment (SOFA) score to analyse respiratory function specifically [17]. The respiratory item of the SOFA score represents the respiratory failure part of the SOFA score. It is inversely correlated to the PaO2/FiO2 ratio, so the lower the PaO2/FiO2 ratio, the higher the respiratory SOFA (SOFA-resp). We applied the SpO2/FiO2 (SF) ratio with a nonlinear imputation from the PaO2/FiO2 (PF) ratio when blood gas were unavailable especially for the non-ICU patients [18]. Worsening of respiratory (SOFA-resp worsening) status was defined as a higher respiratory SOFA at day 5 than at day 1 (if patients died before day 5 respiratory status was considered worsened and, if patient was discharged alive before day 5, the last available respiratory SOFA was used to define worsening). Neutropenia was defined as a neutrophil count lower than 0.5 G/L. Empirical antibiotic therapy, ventilation support and other life-supporting treatments were used according to published guidelines and recent data in all patients [19–21]. Invasive fungal infection especially invasive pulmonary aspergillosis was diagnosed using the most recent European Organization for Research and Treatment of Cancer/Invasive Fungal Infections Cooperative Group and the National Institute of Allergy and Infectious Diseases Mycoses Study Group (EORTC/MSG) consensus group definitions [22,23]. Viral pneumonia was diagnosed if a virus was detected by viral culture or PCR in bronchoalveolar lavage (BAL) fluid, blood or nasopharyngeal specimens with clinical features consistent with viral pneumonia. Hospital and ventilator-associated pneumonia (HAP/VAP) were diagnosed in accordance with the Clinical Practice Guidelines [24]. In addition, a pulmonary co-infection has been defined as a microbiologically confirmed bacterial, viral or fungal pulmonary infection co-occurring at the same time as a documented PJP. All CT were retrospectively analysed. Bilateral and diffuse ground-glass opacities (GGO), predilection for the lung’s upper lobes with normal subpleural lung parenchyma and nodules were considered as typical findings. Conversely, pleural effusion and focal consolidation were considered as atypical findings [25,26].

**Table 1:** Baseline patients characteristics.

**Clinical features**	**Total n = 133**
Male, n (%)	100 (75.2%)
Age, (years), median IQR	64.9 [56.61–73.03]
**Underlying disease, n (%)**	
Haematological malignancies	106 (79.6%)
Lymphoma	42 (31.6%)
Acute lymphoblastic leukemia	16 (12%)
Myeloma	10 (7.5%)
Others	38 (28.6%)
Allogeneic HSCT	17 (12.8%)
Recent neutropenia (within the past week)	7 (5.3%)
Solid tumor	27 (20.3%)
**Exposure to chemotherapy**	
Anthracyclin	22 (16.5%)
Cytarabin	13 (9.8%)
Fludarabin	9 (6.8%)
Rituximab	16 (12%)
Cyclosphophamide	21 (15.8%)
Methotrexate	7 (5.2%)
**Respiratory symptoms, n (%)**	
Dyspnea	110 (82.7%)
Fever	132 (99.2%)
Cough	73 (54.8%)
Charlson Comorbidty Index	3 [2–4]
Anti-PJP prophylaxis, n (%)	20 (15%)
PJP score > 3 in hematology patients, n (%)	102 (96.2%)
PJP score > 3 in all cohort, n (%)	129 (97%)
Shock at admission, n (%)	15 (11.3%)
Previous cortico-steroid treatment, n (%)	58 (43.6%)
SOFA day 1	2 [1–4]
SOFA-resp day 1	2 [1–2]
Time from diagnosis suspicion to PJP- treatment (days)	1 [0–2]
Time from symptom onset to PJP-treatment (days)	6 [3–10]
Time between anti-pneumocystis therapy to EACST (days)	1 [0–4]
**Co-infections, n (%)**	65 (48.9%)
Bacterial	34 (25.6%)
IFI	30 (22.5%)
Viral	39 (29.3%)
HSV1-HSV2	18 (13.5%)
**Severity of illness, n*(%)***	
ICU admission	58 (43.6%)
IMV	46 (34.6%)
Orotracheal intubation between day 1 and day 5	35 (26.3%)
VAP, n (%)	23 (17.6%)
Vasopressors	41 (30.8%)
RRT	18 (13.5%)
30-day mortality	32 (24.1%)
1-year mortality	75 (56.4%)

^a^Median (IQR) unless otherwise specified, HSCT = haematopoietic stem cell transplant, PCP = Pneumocystis Jirovecii Pneumonia, PJP-score = Pneumocystis Jirovecii Pneumonia score, SOFA = sequential organ failure assessment score, SOFA-resp = respiratory item of the SOFA score, ACST = adjunctive corticosteroid treatment, IFI = invasive fungal infection, HSV = herpes simplex virus, ICU = intensive care unit, IMV = invasive mechanical ventilation, VAP = Ventilation associated Pneumonia, RRT = Renal replacement therapy.

### Statistical analysis

Data are described as median and interquartile range for quantitative data and count and proportions for qualitative data. Effect of EACST were assessed on several outcomes: survival within the first 30 days and the first year, worsening of respiratory SOFA score, co-infections, and, for the subgroups of patients not intubated within the first 2 days, tracheal intubation within the first 5 days. To consider the possible selection bias in this observational study (since choice of initiating corticosteroids treatment was let at the discretion of the attendant physician) we used an inverse probability weighting propensity score-based analysis. Propensity score (PS) is the probability to receive EACST based on baseline covariates (age, gender, type of malignancy, allogeneic haematopoietic stem cell transplant, SOFA day 1, SOFA-resp day 1 and ICU admission). Inverse probability weighting (IPW) methods, using truncated stabilized weights, was used to reconstruct a pseudo-population in which patients of both groups (early corticosteroids vs late or no corticosteroids) have similar characteristics and comparison of outcomes is meaningful. We used Cox models to compare time to event outcomes (day 30 and day 365 survival) and logistic models to compare binary outcome (respiratory SOFA worsening, day 5 tracheal intubation and coinfection).

Two sensitivity analyses were performed. First, we compared several choices of weights (namely standards, stabilized, in addition to truncated stabilized). Second, we replicated the first analysis (comparing corticosteroids within 2 days to corticosteroids after 2 days or never) on the subset of patients that were not admitted to ICU within the first 2 days of anti-pneumocystis therapy. All tests were two-sided, and P-values of < 0.05 were considered statistically significant.

## Results

### Patient’s characteristics at admission and during hospital stay

**Fig 1:**
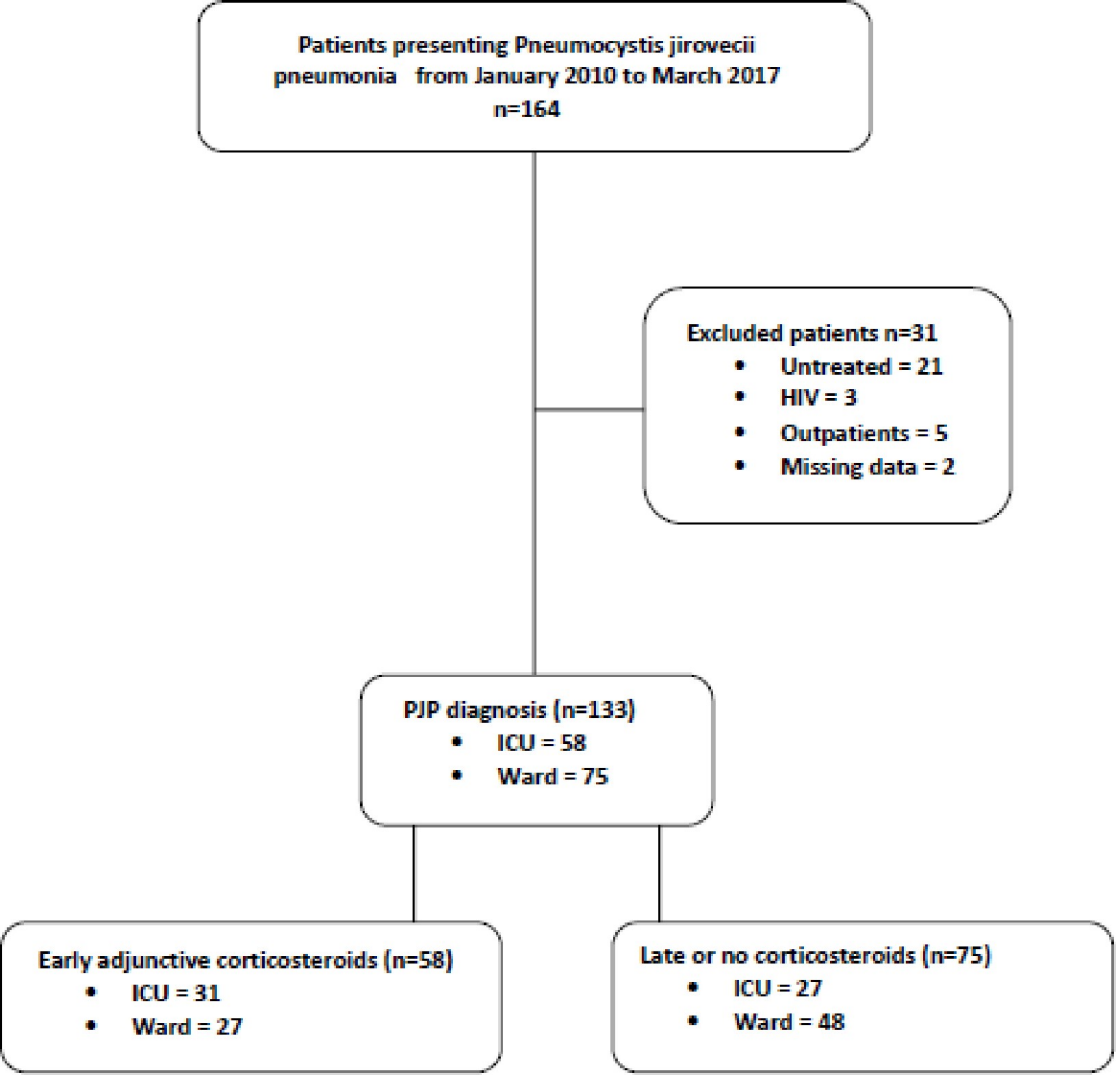
Flow chart.

From 164 patients with PJP diagnosis (66 in ICU and 98 in ward), 31 patients did not meet inclusion criteria and were excluded (two HIV-infected patients. Twenty-one untreated patients, 3 patients with lacking data and 5 patients who were not hospitalized). We included 133 non-HIV patients with confirmed PJP (58 in ICU and 75 in ward) who were admitted between January -1-2010 and March-31-2017 at Institut Paoli Calmettes, Marseille, France **(Fig 1).**

Main clinical characteristics of patients are reported in **Table 1**. All microbiological diagnoses were made using PCR on BAL (8,2%), induced sputum specimen (85%) or nasopharyngeal aspirations (6,8%). In order to confirm the PJP diagnosis, we evaluated PJP-score for the 106 patients with haematological malignancies of the cohort, this score was >3 in 102 haematology patients (96,2%). For all patients in this cohort, PJP score was >3 in 129 patients (97%).

#### Radiological findings

One hundred thirty patients had a CT, 126 (96,9%) had GGO with diffuse GGO for 94 (72,3%) patients and local GGO for 32 (24,6%) patients. Peripheral sparing was found in 17 (13,1%) patients, and crazy paving in 28 (21,5%) patients [27]. Pleural effusion was found in 27 (20,7%) patients and focal condensation in 17 (13,1%) patients. The 3 patients who did not have CT had interstitial opacities at chest radiograph **(Table 2).**

**Table 2:** Computed Tomography (CT) findings.

Radiological characteristics	Total n = 130
Normal CT, n (%)	1 (0.7%)
Ground glass opacities, n (%)	126 (96.9%)
Local ground glass opacities, n (%)	32 (24.6%)
Diffuse ground glass opacities, n (%)	94 (72.3%)
Peripheral sparing, n (%)	17 (13.1%)
Crazy paving, n (%)	28 (21.5%)
Nodules, n (%)	21 (16.1%)
Upper lobe distribution, n (%)	44 (34.8%)
Pleural effusion, n (%)	27 (20.7%)
Focal condensation, n (%)	17 (13.1%)
Cysts, n (%)	1 (0.7%)

### Anti-pneumocystis therapy

All the included patients were treated by anti-pneumocystis therapy, 125 (94%) were treated by SMT, 5 (3,7%) by atovaquone and 3 (2,3%) by pentamidine. Median time (IQR) between symptom onset and initiation of anti-pneumocystis therapy was 6 days [3–10] and median duration (IQR) of anti-pneumocystis therapy was 20 days [19–21].

### Corticosteroids treatment

Among the 133 patients of the study, 88 (66%) received a corticosteroid treatment during the hospital stay. This treatment was considered as EACST in 58 (44%) patients and was delivered in the ICU for 31(53%) patients and in ward for 27 (47%) patients, p = 0.044 (**Fig 1**). The length of stay in ICU for patients treated with EACST was 10 [6.75–31] vs. 21 [7–36] days for other patients, OR = 0.990, CI 95% [0.966–1.015] p = 0.44. The length of stay in hospital for patients treated with EACST was 12 [7–21] vs. 11 [6–23] days for other patients, OR = 0.999, CI95% [0.984–1.015], p = 0.91. Overall, the median (IQR) time between the first day of anti-pneumocystis therapy and adjunctive corticosteroids therapy initiation was 1 day [0–4] (**S1 File**). Seventy-five patients (56%) did not or had a late adjunctive corticosteroids treatment (**Fig 1**).

#### Outcomes

**Fig 2:**
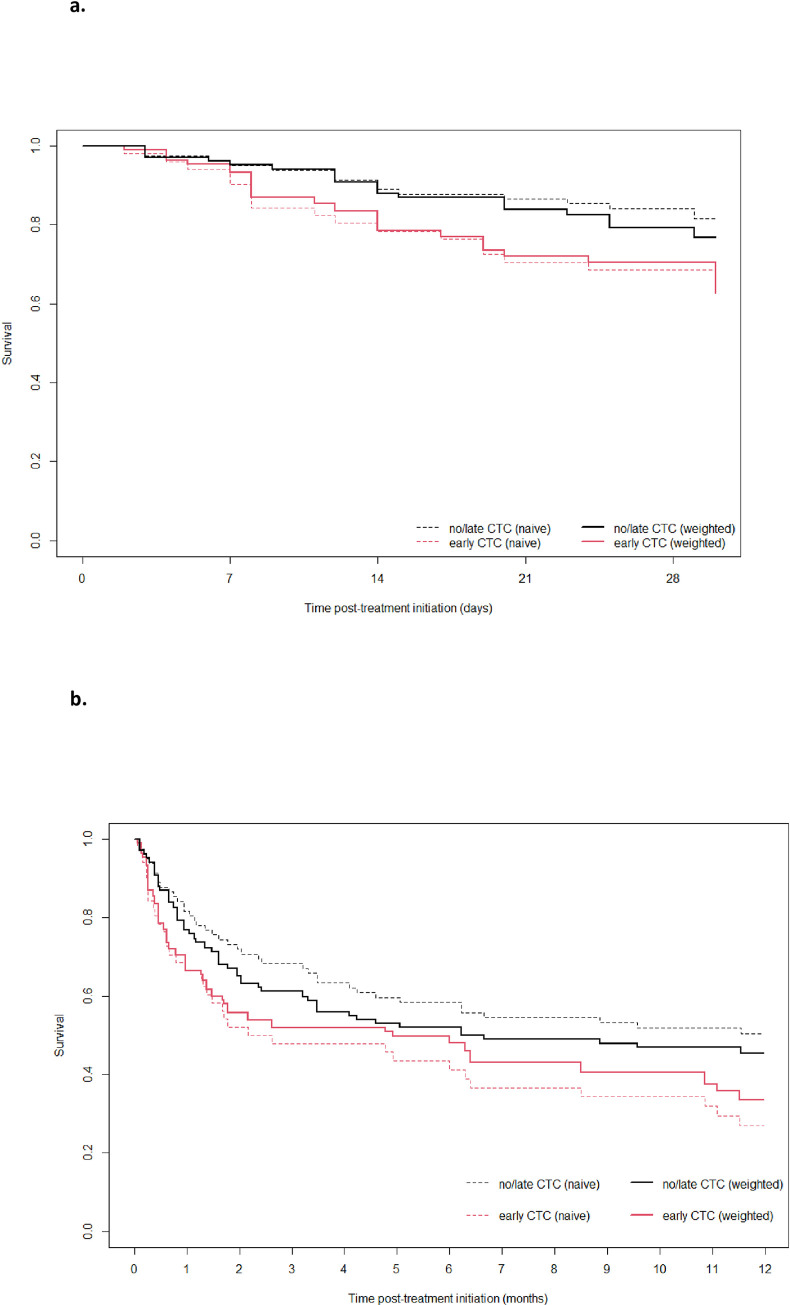
a. Naïve and weighted analysis on 30-days mortality. Effects on 30-days mortality of early corticosteroids therapy (within 48 hours) recipients vs no or late corticosteroids therapy. b. Naïve and weighted analysis on 1-year mortality. Effects on 1-year mortality of early corticosteroids (within 48 hours) therapy vs no or late corticosteroids therapy.

In our study, 30-days and 1-year survival were respectively 24.1% (n = 32) and 56.4% (n = 75) (**Fig 2)**. Regarding patients who received EACST within 48 first hours of anti-pneumocystis therapy initiation, naïve analysis showed there was a significant increase of 30-days (HR = 2.04, CI 95% [1.02–4.09] p = 0.04) and 1-year (HR = 1.85, CI 95% [1.17–2.91], p = 0.008) mortality rate in the EACST groups **(Table 3).** However, using weighted analysis, no significant differences were observed between the EACST group and no or late corticosteroids therapy group on 30-days (HR = 1,45, CI 95% [0.7–3.04], p = 0.32) and 1-year (HR = 1.25, CI 95% [0.75–2.09], p = 0.39) mortality rate **(Table 3).** During the hospital stay 29 (22%) patients developed hospital-acquired pneumonia, 23 (17.6%) developed ventilator-acquired pneumonia. Only one patient developed a pneumothorax related to PJP. After discharge from intensive care, 1 patient was readmitted to intensive care within 48 hours of ICU discharge. After hospital discharge 2 patients were readmitted within one week of hospital discharge.

**Table 3:** Clinical outcomes of early adjunctive corticosteroids therapy after anti-pneumocystis therapy initiation (all patients).

	Naïve survival analysis			IPW survival analysis		
Variable	Effect size	95% CI	p-value	Effect size	95% CI	p-value
**30-days mortality^a^**	2.04	[1.02–4.09]	0.04	1.45	[0.7–3.04]	0.32
**1-year mortality^a^**	1.85	[1.17–2.91]	0.008	1.25	[0.75–2.09]	0.39
**SOFA resp worsening^b^**	1.01	[0.87–1.18]	0.87	0.68	[0.27–1.71]	*0*.*42*
**Intubation day-5^b^**	0.96	[0.84–1.1]	0.56	0.45	[0.12–1.6]	0.22
**Coinfections^b^**	1.1	[0.93–1.31]	0.28	0.89	[0.41–1.91]	0.77

IPW = Inverse Probability Weighting

^a^ Hazard Ratio

^b^ Odd ratio. SOFA = Sequential Organ Failure Assessment Score, SOFA resp = respiratory SOFA.

#### Secondary objectives

Median (IQR) SOFA-resp at day 1 was 2 [1–2] and median (IQR) SOFA at day 1 was 2 [1–4] **(Table 1).** Naïve and weighted analysis did not show a significant difference between the EACST group and no or late corticosteroids therapy group on the SOFA-resp worsening (HR 0,68, CI 95% [0.27:1.71], p = 0,42) between day-1 and day-5 **(Table 3).** Similarly, naïve and weighted analysis did not find significant difference between the EACST group and no or late corticosteroids therapy group regarding need of tracheal intubation at day-5 (OR = 0,45, CI 95% [0,12–1,6] p = 0,22) and concerning occurrence of coinfections (OR = 0,89, CI 95% [0,41–1,91] p = 0,77) **(Table 3).** In fact, 51.7% (n = 30) of patients who were treated with EACST presented a co-infection vs 46.7% (n = 35) in other patients.

#### Sub-group sensitivity analysis

Finally, naïve and weighted sensitivity analysis in sub-group of non-ICU patients with EACST within the first 48 hours after anti-pneumocystis treatment initiation did not show significant difference concerning the 30-days (HR = 2,69, CI 95% [0.84–8.61], p = 0.09) and 1-year (HR = 1,41, CI 95%[0,71–2,8], p = 0,32) mortality rate, SOFA-resp worsening, need of tracheal intubation at day 5 and occurrence of coinfections **(S2 File).**

## Discussion

This retrospective study did not demonstrate benefit associated with EACST on 30-day and 1-year mortality rates in non-HIV oncology or haematology PJP-patients admitted in ICU and/or ward. No significant differences were observed regarding respiratory evolution between day-1 and day-5, need of tracheal intubation at day 5 and occurrence of coinfections. Similar results were found in sub-group sensitivity analysis of non-ICU patients.

PJP is associated with a high mortality rate in patients with impaired cellular immunity as well as in patients with solid tumour (mostly when using longtermly corticosteroids regimen [28,29]) and remain an important cause of acute respiratory failure in patients admitted in ICU [30]. While the use of adjunctive corticosteroids showed an important decrease of mortality in HIV-positive patients with PJP [10], efficacy has not been demonstrated so far for HIV-negative patients with PJP. Although the incidence of PJP is increasing among the non-HIV immunocompromised patient population [4,31], only few studies have assessed the prognosis impact of EACST. Pareja et al in 1998 demonstrated a shorter duration both of mechanical ventilation and ICU length of stay among the corticosteroids recipients but no clinical benefit on mortality [32]. Two retrospective analyses with small population did not demonstrate any clinical benefit of corticosteroids on mortality [33,34]. In the same way, one recent retrospective study of 323 patients have shown that administration of corticosteroids within 48 first hours of anti-pneumocystis therapy was not associated with any clinical benefit in short term respiratory outcomes, need for ICU admission or tracheal intubation, survival or hospital length of stay among PJP-related patients without HIV [35]. In contrast, a retrospective study of 129 ICU-patients revealed that high dose corticosteroids therapy was associated with increased mortality in non-HIV patients with PJP. Interestingly, the authors underline that this increase was not related to higher rate of ICU-acquired infections [36]. Finally, a meta-analysis based on 6 retrospective studies did not demonstrate an association between corticosteroids therapy and survival [37].

The pathophysiology and the immune host response to infection of PJP differs between patients with and without HIV infection [6,31,38–40] and might explain discrepancies regarding therapeutic effect of corticosteroid for the two populations of patients. Prior studies reported that HIV-infected patients with PJP present a lower neutrophils count [41] contrasting with a higher eosinophils count [42] in the BAL as compared with non-HIV PJP immunocompromised patients. Thus, the increase of neutrophils count in the BAL in the non-HIV patients correlates with poorer oxygenation and poorer patient survival [41]. It has been shown that the lysis of pneumocystis pathogen in the lungs after anti-pneumocystis therapy initiation also participates to the pro-inflammatory response in this context [43]. A recent study has shown that BAL fluid cytology profile consistent with alveolitis (> 10% lymphocytes, > 5% neutrophils, and presence of activated macrophages) is associated with less severe PJP and lower 90-day mortality [44]. Taking together these studies suggest that a corticosteroid treatment might have a better clinical effect on eosinophilic alveolitis in HIV-patients than on neutrophilic alveolitis in non-HIV patients [42]. Nevertheless, corticosteroids are frequently associated with adverse effects. Although corticosteroids therapy seems to be associated with an increased risk of viral reactivation and bacterial infection in non-HIV immunocompromised patients [45,46], EACST was not associated with increased risk of co-infections in our study.

In our study EACST was preferentially administrated in more severely ill patient. In fact, 53% of EACST patients received treatment in ICU as compared with 47% in ward. This trend might be due to the consensus recommendations regarding in HIV-infected patients [12] and the lack of robust recommendations for non-HIV population. In order to minimize such bias, we used an inverse probability weighted analysis to adjust the baseline clinical severity of the patients. As expected, naïve analysis showed an increased mortality associated with the EACST, but when using weighted analysis, no significant differences were found. In agreement with the previous retrospective studies, the use of EACST in our study did not demonstrate overall survival benefit. In our study, 30-days mortality was 24,1% which correlates with the data previously published in other retrospective studies. Indeed, Wieruszewski et al [35] and Lemiale et al [36] reported 30-days ward mortality rate of 22,9% and ICU 30-days mortality rate of 26% respectively. To our knowledge, our study is the only one to date to assess long-term mortality among the non-HIV onco-haematology PJP-patients in which 1-year mortality rate was 56,4%.

Our study has several limitations. First, due to the retrospective design of the study, corticosteroid therapy was not standardized during the study period. Duration of treatment and daily dose were let at the discretion of the medical team and might have impact on results. Second, diagnosis of PJP was made only using PCR results and classic clinical criteria without using conventional staining or immunofluorescence [47]. However, in order to confirm the diagnosis of PJP and to exclude the cases of colonization, we used the PJP-score reported in Azoulay et al study [15]. In our study, 102 haematological patients (96,2%) and 129 patients (97%) of all the cohort had a PJP score > 3. Four patients had a PJP-score < 3, due to the use of vasopressor at the time of tracheal intubation. Our results are in concordance with PJP-score and make our PJP diagnoses more robust. Fourth, severity of respiratory failure in non-ICU patients might be underestimated. In fact, for those patients, blood gases were often unavailable because of no arterial line. In order to use the respiratory component of the SOFA score (which was originally developed to utilize the PF ratio), unlike Wieruszewski et al study [35] who used a linear imputation of PF ratio from SF ratio, we applied a nonlinear imputation of PF ratio from SF ratio which seems to be more accurate specifically for moderate to severe hypoxemia [18]. Last, although we used a stabilized and weighted inverse probability weighting analysis to adjust the clinical severity of patients at admission, it might persist undetectable confounding factors.

## Conclusion

In this retrospective study, EACST within 48 hours of anti-pneumocystis therapy was not associated with a decrease of 30-day and 1-year mortalities in non-HIV onco-haematology PJP-patients. Moreover, EACST provides no benefit regarding respiratory evolution and need of tracheal intubation. Finally, EACST seems not to be associated with increased risk of co-infection. The ongoing blinded randomized controlled trial (NCT02944045) may provide valuable insights regarding the interest of EACST in this particular situation.

## Supporting information

S1 File Corticosteroids treatment characteristics. (DOCX)

S2 File Effect of early adjunctive corticosteroid therapy vs no or late corticosteroid therapy in patients not admitted in the ICU within the first 2 days of anti-PJP treatment. (DOCX)

## Abbreviations

BAL: Bronchoalveolar lavage
CT: Computed tomography
EACST: Early adjunctive corticosteroid therapy
GGO: Ground glass opacity
HIV: Human immunodeficiency virus
HSCT: Haematopoietic stem cell transplant
HSV: Herpes Simplex Virus
ICU: Intensive care unit
IFI: Invasive fungal infection
IMV: Invasive mechanical ventilation
IPW: Inverse Probability Weighting
IQR: Interquartile range
PCR: Polymerase chain reaction
PJP: Pneumocystis Jirovecii pneumonia
PS: Propension score
RRT: Renal replacement therapy
SMT: Sulfamethoxazole/Trimethoprim
SOFA-resp: respiratory item of the SOFA score
SOFA: Sequential organ failure assessment score
VAP: Ventilator-associated pneumonia
