# Supplementary material for: Prognostic impact of early adjunctive corticosteroid therapy in non-HIV oncology or haematology patients with Pneumocystis jirovecii pneumonia: A propensity score analysis
Source: PLoS One. 2021 Apr 22;16(4):e0250611. doi: 10.1371/journal.pone.0250611 (PMC8061944; doi:10.1371/journal.pone.0250611)
Supplement: S1 File — (DOCX) [file pone.0250611.s001.docx]

**Supplementary Material 1. Corticosteroids treatment characteristics**

| Characteristics^a^ | All corticosteroids recipients n=88 |
| --- | --- |
| Time to corticosteroids initiation (days)^b^ | 1.0 [0 - 4] |
| Daily corticosteroids dose (mg/kg prednisone equivalent) | 0.8 [0.57 - 1.25] |
| Duration of corticosteroids treatment (days) | 12.5 [6.75 - 19] |

*^a^Median IQR, ^b^since anti-pneumocystis therapy initiation.*

*.*
