# Supplementary material for: Prognostic impact of early adjunctive corticosteroid therapy in non-HIV oncology or haematology patients with Pneumocystis jirovecii pneumonia: A propensity score analysis
Source: PLoS One. 2021 Apr 22;16(4):e0250611. doi: 10.1371/journal.pone.0250611 (PMC8061944; doi:10.1371/journal.pone.0250611)
Supplement: S2 File — (DOCX) [file pone.0250611.s002.docx]

**Supplementary Material** 2**. Effect of early adjunctive corticosteroid therapy vs no or late corticosteroid** **therapy in patients not admitted in the ICU within the first 2 days of anti-PJP treatment**

| Variable | Effect size | 95% CI | p-value | Effect size | 95% CI | p-value |
| --- | --- | --- | --- | --- | --- | --- |
| 30-days mortality^a^ | 3.78 | [1.23 - 11.56] | 0.02 | 2.63 | [0.82 - 8.48] | 0.11 |
| 1-year mortality^a^ | 1.83 | [0.97 - 3.46] | 0.06 | 1.38 | [0.69 - 2.77] | 0.36 |
| SOFA resp worsening^b^ | 0.99 | [0.83 - 1.18] | 0.94 | 0.85 | [0.22 - 3.23] | 0.81 |
| Intubation day-5^b^ | 1 | [0.92 - 1.09] | 0.94 | 0.50 | [0.04 - 5.86] | 0.58 |
| Coinfections^b^ | 1.07 | [0.87 - 1.33] | 0.52 | 1.28 | [0.46 - 3.57] | 0.64 |

**Naïve survival analysis IPW survival analysis**

*IPW= Inverse Probability Weighting, ^a^ Hazard Ratio, ^b^ Odd ratio. SOFA = Sequential Organ Failure Assessment Score, SOFA resp= respiratory SOFA.*
